# Temporal Changes in the Climatic Predictors of Hatching Success in Leach's Storm‐Petrels

**DOI:** 10.1002/ece3.74644

**Published:** 2026-11-02

**Authors:** Ingrid L. Pollet, Robert A. Ronconi, Robert A. Mauck, Patricia L. Jones

**Affiliations:** ^1^ Biology Department Acadia University Wolfville NS Canada; ^2^ Canadian Wildlife Service Environment and Climate Chance Canada Dartmouth NS Canada; ^3^ Biology Department Kenyon College Gambier Ohio USA; ^4^ Biology Department Bowdoin College Brunswick Maine USA

**Keywords:** annual global mean temperature, Atlantic multidecadal oscillation, Atlantic Ocean, *Hydrobates leucorhous*, reproductive success, seabird

## Abstract

Climate change is rapidly altering ecosystems worldwide and can modify the demographic rates of long‐lived vertebrates. For seabirds, which rely on ocean productivity, reproductive success is particularly sensitive to climatic variability. Identifying the scale and stability of climate drivers is therefore critical for predicting population dynamics. Previous work on Leach's storm‐petrels (*Hydrobates leucorhous*) breeding on Kent Island, New Brunswick, showed that Annual Global Mean Temperature (AGMT) best explained hatching success between 1955 and 2010, with a nonlinear response peaking in the late 1980s. Here, we extend this dataset through 2024 to test whether AGMT remains a reliable predictor given the recent rise of ocean temperatures. Using mixed‐effects models, we compared hatching success with multiple climatic indices at regional, basin, and global scales. While AGMT retained some explanatory power, the best performing model for predicting variation in hatching success for the historical dataset (1955–2010) also included the Southern Oscillation Index (SOI), which was not evaluated in the original analyses, and the Atlantic Multidecadal Oscillation (AMO) for the full dataset (1955–2024). Different pathways may be involved: SOI could reflect non‐breeding conditions and have a carry‐over effect, whereas AMO could change prey availability and foraging conditions during egg formation and incubation. The relationships of hatching success with environmental indices changed over time. These results provide evidence that climate–biology relationships can be temporally dynamic, with the effects of some climatic drivers on hatching success changing over the study period. The AMO has also been linked to variation in adult survival for this population; its emerging importance for hatching success implies a compounded demographic impact with consequences for the population vulnerability.

## Graphical abstract

Long‐term monitoring of Leach's storm‐petrels reveals that climatic drivers of hatching success are not stable through time. Global temperature once best explained reproductive success, but basin‐scale variability now plays a stronger role, demonstrating that climate–demography relationships can shift as the climate system changes.

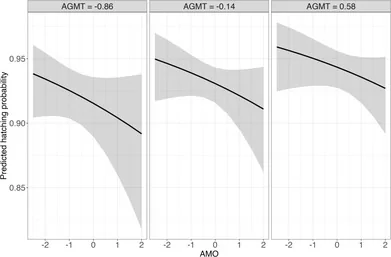

## 1 Introduction

Climate change has measurable effects on species demography, including for long‐lived marine vertebrates (Sydeman et al. 2015; Dias et al. 2019; Killeen et al. 2025), with recent work showing that seasonal ocean warming can differentially affect seabird reproduction and survival across the annual cycle (Layton‐Matthews et al. 2025). These effects may manifest gradually, such as through shifting phenologies or declining reproductive output, and are best detected using long‐term biological datasets (Gordo and Sanz 2005; Jenouvrier et al. 2005). In seabirds, climate variability can affect reproduction indirectly through changes in oceanographic conditions and marine productivity (Grémillet and Boulinier 2009). In turn, marine productivity can alter prey availability and the foraging conditions experienced by breeding adults (Fennie et al. 2022). Identifying the climatic drivers associated with variation in reproduction success is crucial for understanding population dynamics (Sydeman et al. 2015; Pistorius et al. 2023). Previous studies have linked seabird demographic rates to regional oceanographic indices, such as the North Atlantic Oscillation (NAO) or the Atlantic Multidecadal Oscillation (AMO), with mixed support (Frederiksen et al. 2004; Oliveira et al. 2023; Pollet et al. 2025).

Climate can influence seabird demographic rates through several pathways, operating at different spatial and temporal scales. For example, changes in sea temperature, circulation, and wind conditions can modify primary productivity and thus the abundance, distribution, and accessibility of prey for seabirds (Raymond et al. 2010). When this occurs during the breeding season, this could affect adult foraging effort and body conditions and in turn influence egg production, incubation behavior, and chick provisioning (Rindorf et al. 2000; Fayet et al. 2020). Alternatively, when those difficult foraging conditions occur in the non‐breeding season, they can have a carry‐over effect for the subsequent breeding season (Sorensen et al. 2009; Seyer et al. 2023).

Leach's storm‐petrels (*Hydrobates leucorhous*) are small (~45 g) seabirds breeding across the northern hemisphere (Pollet, Bond, et al. 2019), with declining populations (BirdLife International 2018; COSEWIC 2020). On Kent Island, New Brunswick, Canada, Mauck et al. (2018) showed that Annual Global Mean Temperature (AGMT) was the strongest predictor of hatching success across 56 years (1955–2010). No other variable tested, including local temperature, sea surface temperature anomaly, and the NAO, could explain the patterns as effectively as AGMT. Their analysis revealed a nonlinear relationship between AGMT, which has had a positive trend since 1950, and hatching success. Reproductive output increased with AGMT up to a threshold (0.41, reached around 1988), after which it declined while AGMT continued to increase. These findings suggest that global‐scale climate conditions, not just regional or colony‐level factors, may influence breeding performance in this pelagic seabird. A recent study on this population (1955–2023) reveals that another global index, the AMO, partly explained the survival of Leach's storm‐petrels on Kent Island (Pollet et al. 2025). In that study, the effects of AGMT and NAO were not as strong as those of AMO.

Since the 2018 study, which used data from 1955 to 2010 (Mauck et al. 2018), global temperatures have continued to rise, and the period 2010–2024 includes the warmest years on record (Arguez et al. 2020). In particular, the Gulf of Maine and the Scotian Shelf have seen very rapid warming in recent decades (Pershing et al. 2015; Seidov et al. 2021) and these waters encompass the foraging areas for Leach's storm‐petrels breeding on Kent Island. Heat waves produced by these warm temperatures can have drastic negative impacts on the reproductive success of seabirds (Montevecchi et al. 2020; Piatt et al. 2020). Whether AGMT remains a predictor of reproductive output in Leach's storm‐petrels under this new climatic regime remains unknown. This is particularly important considering that the sensitivity of demographic traits to climate may not remain stationary through time (Jenouvrier 2013).

In this study we extended the dataset presented by Mauck et al. (2018) by analyzing hatching success through 2024 from the same colony. We tested whether climate‐demography relationships identified under historical conditions continued as the climate warmed. We expected that the previously reported nonlinear relationship with AGMT would either persist, with hatching success further declining with elevated temperatures, or weaken, if the relationship between global temperature and environmental conditions experienced by breeding Leach's storm‐petrels changed through time. We also evaluated regional and global‐scale climate indices that could influence reproductive success. Specifically, we predicted that changes in North Atlantic conditions, represented by the AMO and NAO could influence hatching success through changes in prey availability influenced by wind and temperature regimes. We hypothesize that high sea surface temperature would negatively impact hatching success. We further considered the Southern Oscillation Index (SOI) because climate conditions experienced during migration may affect birds during the breeding season. Positive SOI values strengthen upwellings and should benefit foraging conditions. Finally, for the period for which daily SST data are available (1982–2024), we tested whether heatwaves within the foraging area during incubation were associated with reduced hatching success. By updating this long‐term dataset, we assessed the stability of climate‐demography relationships in a long‐lived seabird with environmentally sensitive reproduction.

## 2 Methods

### 2.1 Field Site and Species

Kent Island, New Brunswick (44°35′ N, 66°45′ W) is an 85‐ha island in the Grand Manan Archipelago, located at the mouth of the Bay of Fundy (Figure 1). The island is the breeding ground of ~21,600 pairs of Leach's storm‐petrels (D'Entremont et al. 2020). Leach's storm petrels are long‐lived (up to ~37 years) and lay one egg per year, with an incubation period of 40–44 days. They show strong mate and burrow fidelity (Morse and Kress 1984). Yearly monitoring of all the burrows within a confined study area started in 1955, with the intention of banding breeding adult pairs with metal US Fish and Wildlife/Canadian Wildlife Service bands and recording hatch success. During the laying period, burrows were checked daily to determine occupancy and detect newly laid eggs. Once an egg was detected, burrows were checked again every other day starting 38 days later for sign of hatching. Because all known burrows within the study plots were monitored and breeding attempts were detected early on (incubation), hatching success was calculated from the fate of known eggs rather than using an estimator (Johnson 1979). Hatch success was not recorded in 1977 (no field work that year), as well as in 2020 and 2021 (limited presence on the island due to the COVID‐19 pandemic). Permission to handle study animals was authorized under Canadian Wildlife Permits #SC2789, #02109, and Animal Care and Use Permits #ROC2008‐2014, #IACUC2023‐0014.

**FIGURE 1:**
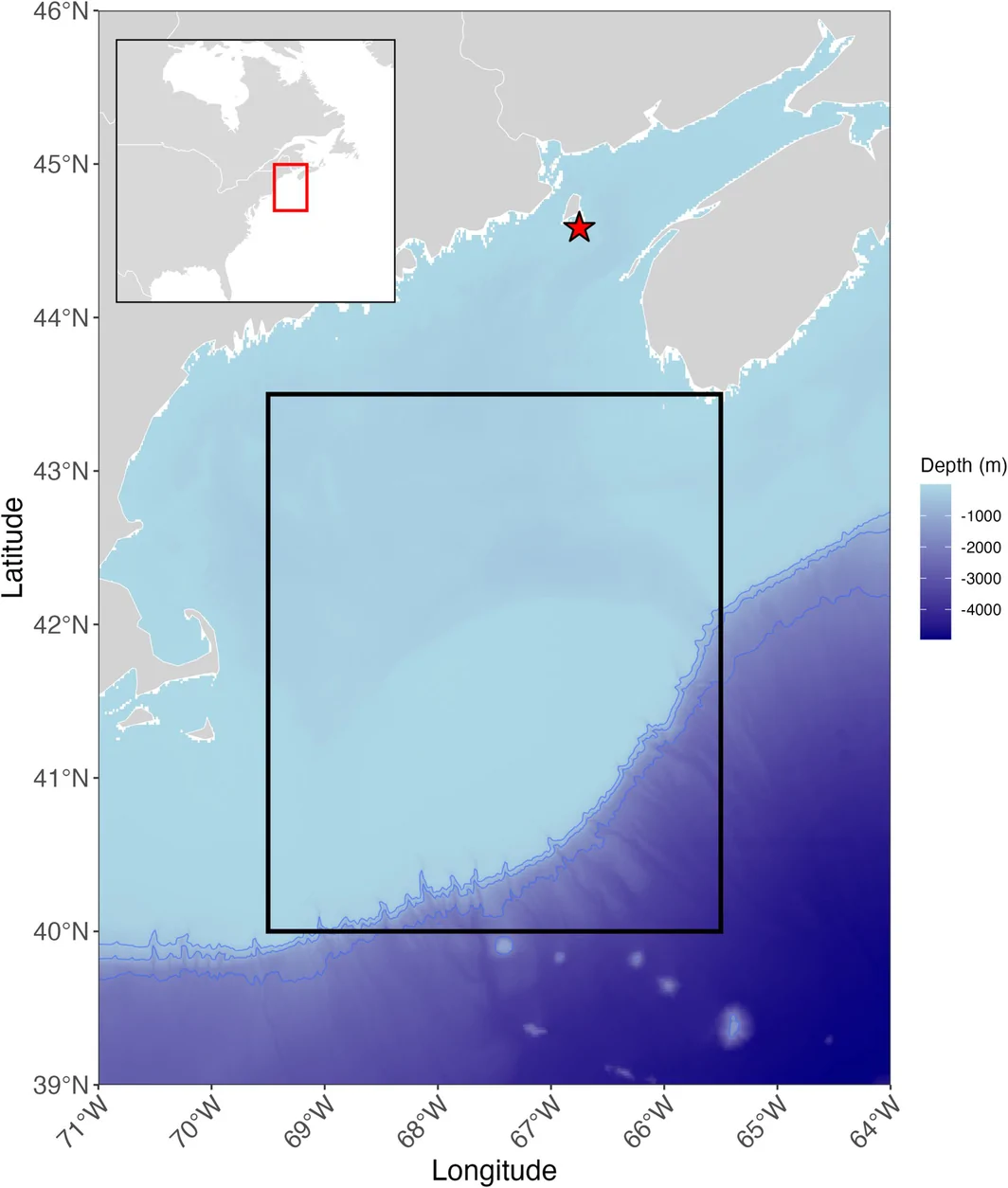
Location of Kent Island, New Brunswick (red star) and area bound to extract Sea Surface Temperatures (SST) for the heat wave data (black rectangle), based on the core foraging area of Leach's storm‐petrels from that colony. Contour lines are drawn at the depth of 50, 100, 200, 500, 1000, and 2000 m.

### 2.2 Environmental Variables

To match analysis from Mauck et al. (2018), we used as a predictor for the full dataset (1955–2024): the Annual Global Mean Temperature (AGMT), a global index derived from the combination of marine and terrestrial temperatures (Hansen et al. 2006). AGMT values were downloaded from the National Oceanic and Atmospheric Administration (NOAA) site (https://www.ncei.noaa.gov/access/monitoring/climate‐at‐a‐glance/global/time‐series).

We also used the Atlantic Multidecadal Oscillation (AMO), which represents detrended Sea Surface Temperature (SST) anomalies averaged over the North Atlantic, whereby positive and negative values correspond to warmer and colder SST, respectively, than the long‐term mean. We used the unsmoothed detrended values from the Kaplan SST V2 (https://psl.noaa.gov/data/timeseries/AMO/), and summer (May, June, July; AMOs) and annual (AMOy) measures. The detrended AMO is intended to represent interannual variability, independent of the long‐term warming trend. We used the North Atlantic Oscillation (NAO) Index (https://www.ncei.noaa.gov/access/monitoring/nao/), known to influence various breeding parameters of seabirds in the Atlantic Ocean (Sandvik et al. 2005; Pereira et al. 2020). We also used the Southern Oscillation Index (SOI) (https://www.ncei.noaa.gov/access/monitoring/enso/soi), an indicator of air pressure between Tahiti, French Polynesia and Darwin, Australia, with effects beyond the Pacific Ocean (Tavares et al. 2020; Morley et al. 2025). Sustained negative SOI values signal El Niño phenomena while positive values signal La Niña phenomena. We used the yearly (SOIy) and the winter (December, January, February; SOIw) values to reflect the annual period when some Leach's storm‐petrels are in the southern hemisphere (Pollet, Bond, et al. 2019). Finally, the Sea Surface Temperature (SST) with a daily 0.25° × 0.25° resolution (https://coastwatch.pfeg.noaa.gov/erddap) was obtained for May, June, and July (1982–2024) within the −69.5° E, 40° N, −65.5° E, 43.5° N boundaries (Figure 1) to calculate a Heat Wave Index (HWI, see below). These boundaries represent the core foraging area for Leach's storm‐petrels from Kent Island during incubation (Hedd et al. 2018; Mauck et al. 2022). These variables were chosen to represent climatic processes operating at global, basin‐wide, regional, and local oceanographic scales that could potentially influence hatching success. Other local factors, such as precipitation, predation pressure, or contaminant burden could also affect the reproductive output, but were not included in the models since they have not been recorded systematically at relevant temporal or spatial (local) scales in this study.

### 2.3 Statistical Analysis

All statistical analyses were performed using R version 4.4.1 (R Core Team 2024) within R Studio version 2024.04.2 + 764. Using the daily SST average values across the boundaries mentioned above, we calculated a daily temperature threshold for the 1982–2024 period using “ts2clm” from the “heatwaveR” package (Schlegel and Smit 2018). We then estimated marine heat wave events using “detect_event” that follow the Hobday et al. (2016) definition of a period of at least five consecutive days when SST exceed the corresponding climatological 90th percentile threshold. We extracted the intensity (°C) and duration (days) of each heat wave in this region. The yearly HWI was calculated as the sum of the intensity × duration of all heat waves for that period.

$${\mathrm{HWI}}_{i}=\sum {\text{Intensity}}_{\mathrm{ij}}\times {\text{Duration}}_{\mathrm{ij}}$$

*i* = year, *j* = specific heat wave in year_*i*_.

The first heat wave in the region was in 1999, but the HWI amplified starting in 2012 and reached a current maximum in 2023 (Figure 2a).

**FIGURE 2:**
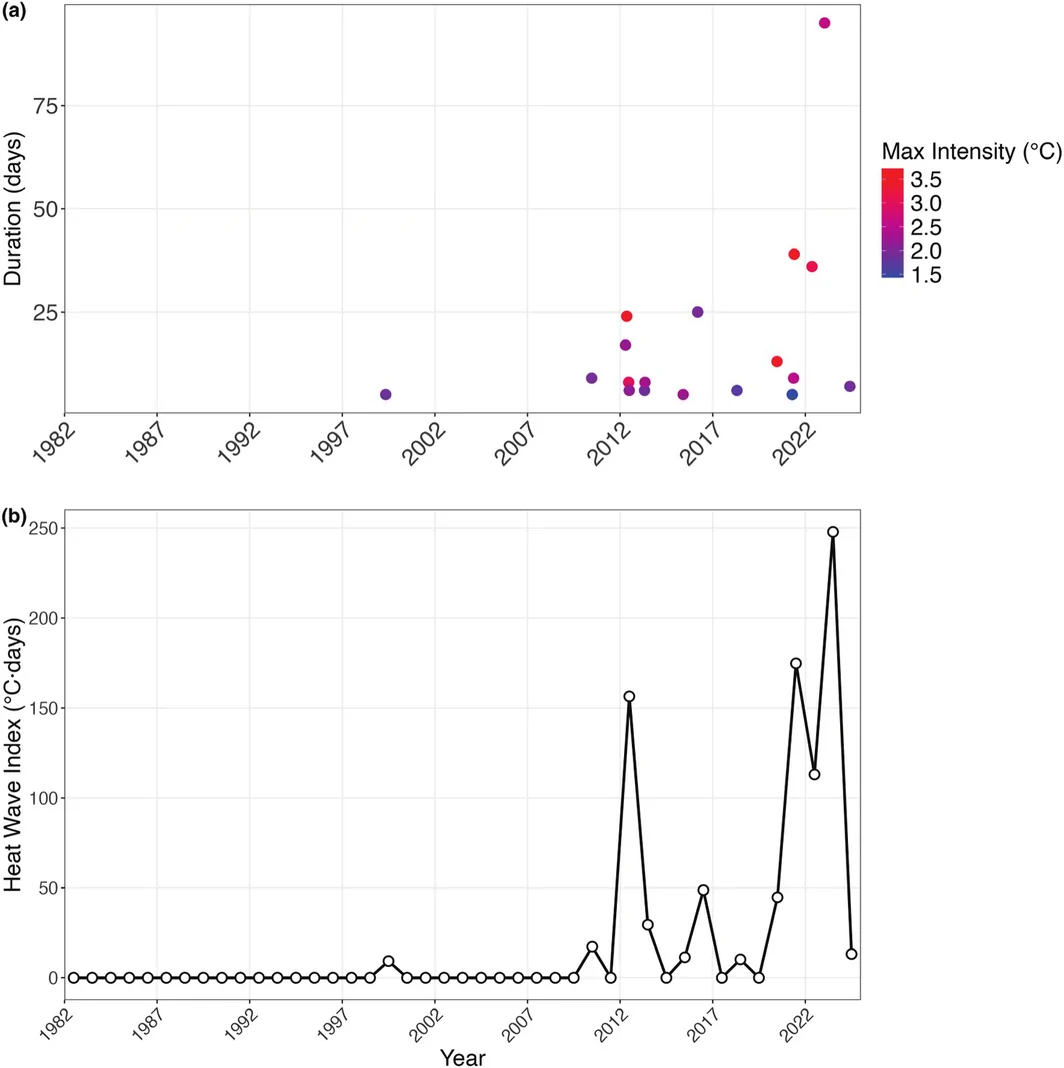
Intensity and duration of heat waves (a) and annual heat wave index (b) calculated as the sum of the intensity and duration of heat waves in May, June, and July in the summer bound area. Marine heatwaves were defined as periods when sea‐surface‐temperature (SST) exceeded the seasonally varying 90th‐percentile threshold. The intensity represents the maximum SST anomaly (°C) relative to the seasonal mean. Anomalies in the region have been as high as 5°C (https://www.nesdis.noaa.gov/news/warm‐sea‐surface‐temperatures‐the‐western‐north‐atlantic).

Using a similar approach to Mauck et al. (2018), we first created intrinsic models of hatch success as a binomial response variable, without environmental variables. The best‐supported intrinsic model was then used as the null model against which the models incorporating environmental variables were compared. We fitted binomial generalized mixed‐effect models with the “glmer” function from the “lme4” package (Bates et al. 2015) with a logit link. Fixed effects included breeding experience (BE), defined as the number of years since the bird was first detected on the study plot, and pair bond length (PBL) defined as the number of years since that pair was first detected together. Random effects included year, to account for unmeasured annual variation shared among observations within the same breeding season, as well as individual ID (band number), pair bond ID (combination of both partner's band numbers), and burrow ID (unique number identifying the burrow). These random effects accounted for reapeated observations of individuals, which do not necessarily represent the same biological units over time because mate switching occurs (4.3% annually; Pollet et al. 2026), and individuals may skip breeding seasons (personal observations). In total, we evaluated 45 models. Assumptions of hatching success models were verified by assessing the over‐dispersion using the “RVAideMemoire” package (Hervé 2017). The best supported intrinsic model was assessed for singularity using “isSingular” from the “lme4” package (Bates et al. 2015) and was used as the baseline for testing climatic variables. We then added to this null model various scaled climatic indices in linear or quadratic forms, independently, or in combination.

Models were evaluated over three overlapping periods, each addressing a distinct question. The 1955–2010 period matched the temporal analysis performed by Mauck et al. (2018), allowing comparison with results from this historical dataset. The 1955–2024 period used the complete available time series to assess whether these relationships remained supported after extending the historical dataset, considering the pattern of climate change in that new period. Finally, analysis including heatwaves was constrained to the 1982–2024 period, which represents the longest period for which the heatwave index was available. To assess whether the relationships between predictors and hatching success change through time, we additionally fitted models including interactions between each environmental predictor (SOI, AGMT, AMO, and NAO) and year. In this case, year was included as a continuous, mean‐centered fixed effect.

Model assumptions were assessed by examining collinearity among environmental variables using variance inflation factors (VIFs) and temporal autocorrelation in simulated model residuals using DHARMa. For the temporal autocorrelation, residuals were aggregated by year and assessed using a Durbin‐Watson test. We additionally fitted a sensitivity model in which the year random intercept was replaced by a continuous‐time Ornstein‐Uhlenbeck correlation structure, which accounted for the unequal temporal spacing among sampled years (years 1977, 2020, and 2021 missing). Model support was compared using AIC, and fixed‐effect estimates were compared between models to assess the robustness of inference to temporal structure. Although both year (as a random effect) and climatic predictors varied at the same annual scale, year accounted for residual between‐year variability not explained by the climatic variables, thereby reducing the risk of attributing unexplained annual variation to climate indices. Models were compared using Akaike's information criterion (AIC) with a correction for sample size (AICc; Burnham et al. 2002).

## 3 Results

### 3.1 Intrinsic Models

The best‐supported intrinsic model for each period included breeding experience (BE) and pair bond length (PBL) as fixed effects, with random intercepts for year, pair bond identity, and burrow identity (Table 1; Table S1 for all models). No evidence of overdispersion was detected (dispersion ratio = 0.47, 0.46, and 0.46 for each period, respectively). BE had a positive effect on hatching success whereas PBL was retained as it improved the intrinsic model fit. Hatching success in Leach's storm‐petrels varied over the study period (1955–2024), with marked interannual variability (Figure 3). Lower hatching success in the first 2 years of the study should be interpreted with caution, as data are based on fewer burrows (*n* = 97 and 78) than most years (mean = 423).

**TABLE 1:** Summary of the top performing intrinsic models (without climatic variables) of hatching success of Leach's storm‐petrels on Kent Island, for each period tested (and corresponding observations).

	*K*	AICc	Δ AICc	Wt	Cum
Models for the 1955–2010 period (25,879 obs.)					
PBL + BE + (1|Y) + (1|Br) + (1|Pair)	6	20,584.60	0.00	0.73	0.73
PBL + BE + (1|Y) + (1|Br) + (1|ID) + (1|Pair)	7	20,586.62	2.01	0.27	1.00
PBL + (1|Y) + (1|Br) + (1|ID) + (1|Pair)	6	20,596.75	12.14	0.01	1.00
BE + (1|Y) + (1|Br) + (1|ID) + (1|Pair)	6	22,026.84	1442.24	0.00	1.00
Null	5	22,042.12	1457.52	0.00	1.00
BE + (1|Pair)	3	22,410.77	1826.16	0.00	1.00
Models for the 1955–2024 period (27,955 obs.)					
PBL + BE + (1|Y) + (1|Br) + (1|Pair)	6	22,318.68	0.00	0.73	0.73
PBL + BE + (1|Y) + (1|Br) + (1|ID) + (1|Pair)	7	22,320.68	2.00	0.27	1.00
PBL + (1|Y) + (1|Br) + (1|ID) + (1|Pair)	6	22,330.45	11.77	0.00	1.00
BE + (1|Y) + (1|Br) + (1|ID) + (1|Pair)	6	23,759.71	1441.03	0.00	1.00
Null	5	23,775.39	1456.70	0.00	1.00
BE + (1|Pair)	3	24,201.10	1882.41	0.00	1.00
Models for the 1982–2024 period (18,635 obs.)					
PBL + BE + (1|Y) + (1|Br) + (1|Pair)	6	14,525.41	0.00	0.70	0.70
PBL + BE + (1|Y) + (1|Br) + (1|ID) + (1|Pair)	7	14,527.41	2.00	0.26	0.95
PBL + (1|Y) + (1|Br) + (1|ID) + (1|Pair)	6	14,530.83	5.42	0.05	1.00
BE + (1|Y) + (1|Br) + (1|ID) + (1|Pair)	6	15,212.17	686.76	0.00	1.00
Null	5	15,218.20	692.79	0.00	1.00
BE + (1|Pair)	3	15,475.41	950.00	0.00	1.00

*Note:* See Table S1 for all models.

Abbreviations: Δ AICc, difference between model with lowest AICc and other models; AICc, Akaike's information criterion adjusted for sample size; BE, breeding experience; Br, burrow ID; Cum, Cumulated model weights; ID, individual ID; K, number of parameters; Pair, Pair ID; PBL, pair bond length; Wt, Akaike weight; Y, year.

**FIGURE 3:**
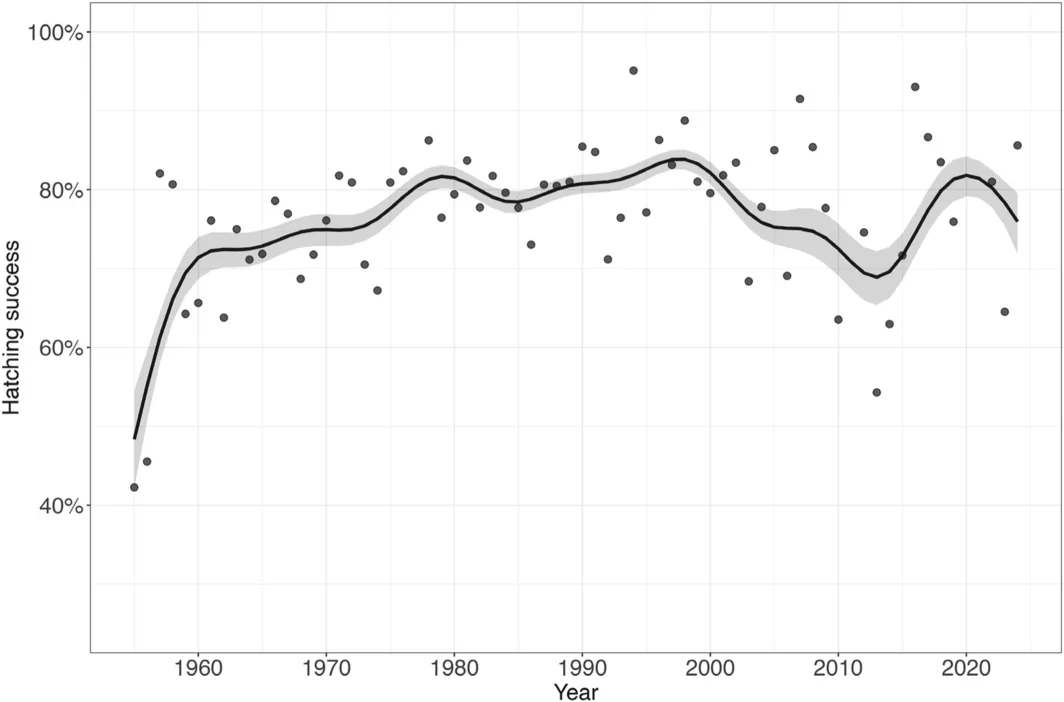
Annual hatching success (points) of Leach's storm‐petrel and a smoothed temporal trend (black line) fitted to annual binomial data with 95% confidence intervals (gray shade).

### 3.2 Models for the 1955–2010 Period

Using the same dataset as Mauck et al. (2018), the model including the combined effect of the quadratic form of the Annual Global Mean Temperature AGMT and the linear form of the yearly Southern Oscillation Index (SOIy) was the top performing model to estimate hatching success (Table 2). This indicates an increase in hatching success with increasing temperature, up to a certain point (AGMT = 0.402), then a decrease in hatching success (quadratic AGMT estimate (±SE) = −0.40 ± 0.16, *Z* = −2.46, *p* = 0.013). For the SOI, hatching success decreased with increasing SOI values (estimate = −0.20 ± 0.07, *Z* = −2.68, *p* = 0.007).

**TABLE 2:** Summary of the top performing models (less than 10 Δ AICc of the top performing model) and null model of hatching success of Leach's storm‐petrels on Kent Island with various environmental variables using three different periods.

	*K*	AICc	Δ AICc	Wt	Cum
Models for the 1955–2010 period					
AGMT^2^ + SOIy	8	20,569.67	0.00	0.51	0.51
AGMT^2^ + SOIy^2^	9	20,570.51	0.85	0.33	0.84
AGMT^2^ + NAOy	8	20,573.58	3.92	0.07	0.91
AGMT^2^ + NAOy^2^	9	20,574.60	4.94	0.04	0.95
AGMT^2^	7	20,574.94	5.27	0.04	0.99
SOIy	7	20,577.66	7.99	0.01	1.00
Null	6	20,584.60	14.95	0.00	1.00
Models for the 1955–2024 period					
AGMT + AMO	7	21,830.36	0.00	0.50	0.50
AGMT^2^ + AMO	8	21,831.21	0.84	0.33	0.83
AMO	6	21,833.24	2.87	0.12	0.95
AMO^2^	7	21,835.19	4.82	0.05	1.00
Null	6	22,318.68	488.32	0.00	1.00
Models for the 1982–2024 period					
AMO	7	14,043.18	0.00	0.41	0.41
AMO + AGMT	8	14,044.01	0.83	0.27	0.68
AMO + HW	8	14,044.88	1.70	0.17	0.85
AMO^2^	8	14,045.17	1.98	0.15	1.00
Null	6	14,525.41	482.23	0.00	1.00

*Note:* For each period, the null model (and each of the models with environmental variables) included breeding experience and pair‐bond length as fixed effects, with random intercepts for year, pair bond ID, and burrow ID (see Table 1). Superscript “2” denotets quadratic form of the parameter.

Abbreviations: Δ AICc, difference between model with lowest AICc and other models; AGMT, Annual Global Mean Temperature; AICc, Akaike's information criterion adjusted for sample size; AMO, Atlantic Multidecadal Oscillation; Cum, Cumulated model weights; HW, Heat Wave; K, number of parameters; NAOy, yearly North Atlantic Oscillation; SOIy, yearly Southern Oscillation Index; Wt, Akaike weight.

### 3.3 Models for the 1955–2024 Period

When taking the full dataset (1955–2024) into consideration, the top performing model to estimate the hatching success of Leach's storm‐petrel included linear AGMT and the Atlantic Multidecadal Oscillation (AMO; Table 2), with hatching success significantly increasing with increasing AGMT values (estimate: 0.30 ± 0.14, *Z* = 2.21, *p* = 0.027) but with little effect of AMO (estimate: −0.13 ± 0.11, *Z* = 1.25, *p* = 0.21; Figure 4). The second‐best model included the quadratic form of AGMT (Δ AICc = 0.84). Collinearity among environmental variables was low. For the top performing model, VIFs were 1.43 for both variables. The year random‐intercept model showed modest positive residual temporal autocorrelation (Durbin‐Watson = 1.46, *p* = 0.027). However, replacing the year random intercept with a continuous‐time Ornstein‐Uhlenbeck correlation structure did not improve model support (AIC = 21,832.22 versus 21,830.36), with fixed‐effect estimates virtually unchanged. The simpler year random‐intercept structure was therefore retained.

**FIGURE 4:**
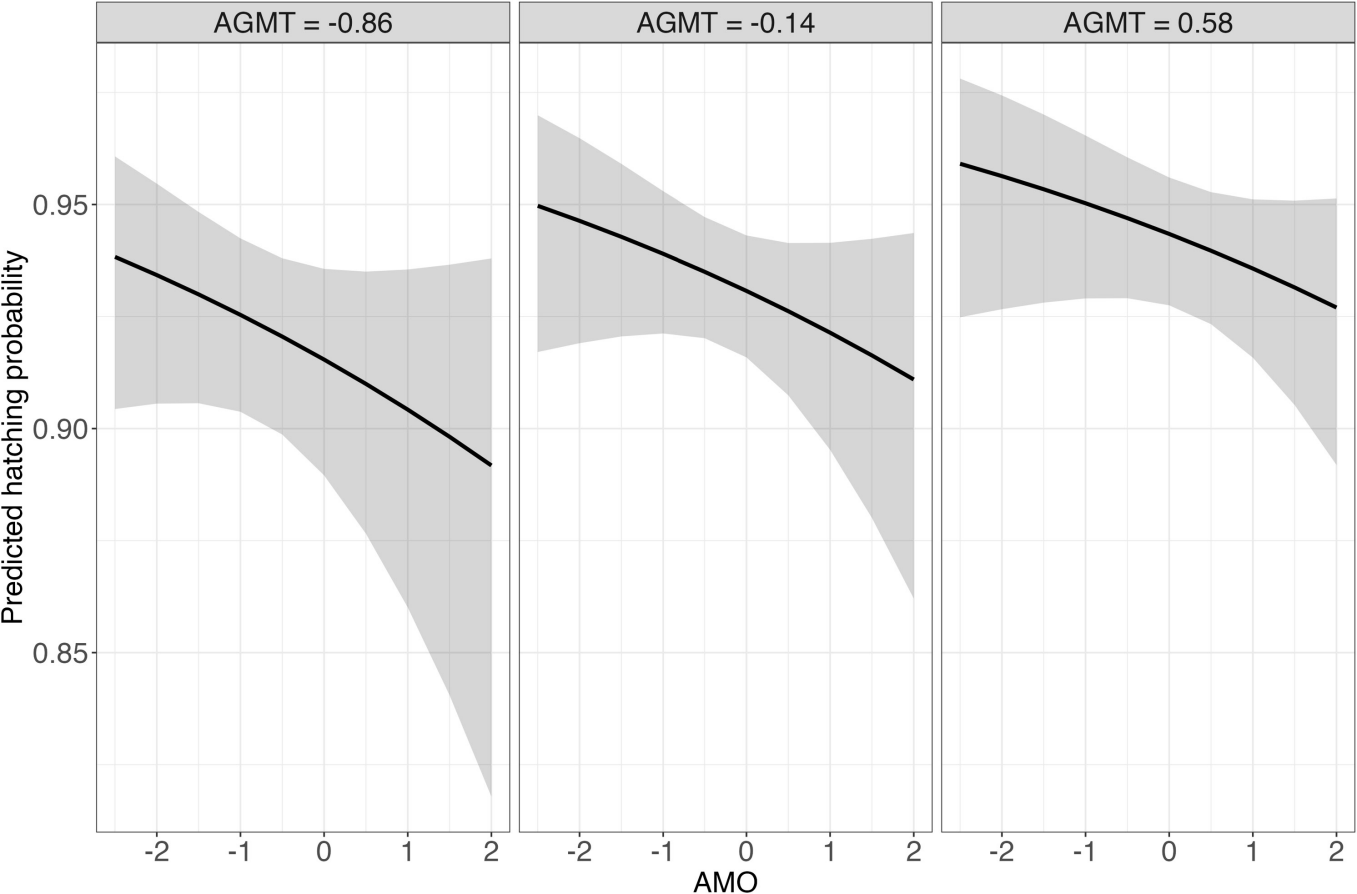
Predicted hatching probability of Leach's storm‐petrels across Atlantic Multidecadal Oscillation (AMO) under different Annual Global Mean Temperature (AGMT) values from a GLMM (binomial logit) controlling for breeding experience and bird identification, pair identification, burrow, and year, using the full dataset (1955–2024).

We tested interactions between year and climatic predictors (both linear and quadratic forms). We found significant temporal changes in the relationships between hatching success and SOI (SOI × Year: *β* = 0.0158 ± 0.0046 SE, *p* < 0.001; SOI^2^ × Year: *β* = 0.0096 ± 0.004 SE, *p* = 0.016) and AGMT (AGMT × Year: *β* = −0.020 ± 0.006 SE, *p* < 0.001; AGMT^2^ × Year: *β* = 0.014 ± 0.0001 SE, *p* < 0.001). However, there was no evidence of that relationship with AMO (*p* = 0.69 and 0.4 for linear and quadratic form) and NAO (*p* = 0.85 and 0.06 for linear and quadratic form).

### 3.4 Models for the 1982–2024 Period

Using the longest possible dataset containing heatwave index (1982–2024, Figure 2b), the best performing model to estimate hatching success of Leach's storm‐petrel included linear AMO as a unique environmental variable (Table 2), and adding the heatwave index did not significantly improve the model (ΔAIC = 1.70, *χ* ^2^ = 0.31, *p* = 0.58).

Variance partitioning of the best‐supported environmental model for each period indicated that most of the latent‐scale residual variation in hatching success occurred at the pair level, with pair ID accounting for 69.7%–71.5% of the variance after accounting for the fixed effects (Table 3). Burrow ID explained a smaller but non‐negligible proportion of the variance (7.6%–7.9%), whereas relatively little residual between‐year variance remained after accounting for the fixed effects, including environmental variables.

**TABLE 3:** Variance partitioning on the latent scale for the best‐supported environmental generalized linear mixed model of hatching success for each period.

	Variance	Percent
Model for the 1955–2010 period (AGMT^2^ + SOIy)		
Pair ID	10.91	69.7
Residual	3.29	21.0
Burrow	1.22	7.8
Year	0.22	1.4
Model for the 1955–2024 period (AGMT + AMO)		
Pair ID	11.22	69.8
Residual	3.29	20.5
Burrow	1.22	7.6
Year	0.35	2.2
Model for the 1982–2024 period (AMO)		
Pair ID	12.36	71.5
Residual	3.29	19.0
Burrow	1.37	7.9
Year	0.26	1.5

## 4 Discussion

The environmental variables receiving the greatest model support for hatching success in Leach's storm‐petrels differed depending on the temporal extent of the dataset. Over the initial period considered in the Mauck et al. (2018) study (1955–2010), variation in hatching success was best explained by a nonlinear relationship with Annual Global Mean Temperature (AGMT) together with the Southern Oscillation Index (SOI), whereas extending the time series through 2024 resulted in greater support for models containing AGMT and the Atlantic Multidecadal Oscillation (AMO). Although the estimated AMO effect was negative, its confidence interval overlapped zero, indicating uncertainty in the strength of this relationship. These results demonstrate that conclusions regarding climate–reproduction relationships can be sensitive to the temporal extent of long‐term demographic datasets. This is particularly relevant under ongoing climate change, because relationships estimated from historical observations may not necessarily provide reliable representations of current demographic responses under increasingly changing environmental conditions.

Over the historical period, the nonlinear relationship between AGMT and hatching success was consistent with that previously identified by Mauck et al. (2018), with hatching success increasing with AGMT to an apparent optimum before declining at higher temperatures. Add in here that it was linear for the full dataset before you enter into the explanation? Such a relationship could reflect the cumulative influence of warming on marine productivity, prey availability, phenological timing, or conditions experienced during breeding. Moderate warming may initially benefit breeding performance, whereas sustained warming may eventually be associated with reduced reproductive performance (Halupka et al. 2023). In Leach's storm‐petrels, deteriorating environmental conditions could increase foraging‐trip duration, reduce adult condition, or increase the frequency of incubation interruptions, ultimately reducing hatching success. Continued collection of information on adult foraging behavior and condition alongside reproductive monitoring would help determine the mechanisms underlying this nonlinear relationship.

The SOI also contributed to the best‐supported model for the historical period, with hatching success declining with increasing SOI values. SOI influences wind conditions, sea‐surface temperatures, and upwelling (Bylhouwer et al. 2013; Infanti and Kirtman 2016), and has been associated with diet, breeding success, and population demography in seabirds (Pistorius et al. 2023). Although ENSO‐related effects on seabirds are most commonly documented in the Pacific, they can extend to species breeding in the Atlantic (Jenouvrier et al. 2009). For Leach's storm‐petrels, one possible pathway for SOI to influence breeding success is through environmental conditions encountered outside the breeding season. Many individuals from this population migrate to the Southern Hemisphere (Pollet, Ronconi, et al. 2019), and environmental conditions experienced during migration and winter could have carry‐over effects on subsequent reproduction (Desprez et al. 2018). Linking individual non‐breeding movements and environmental exposure to subsequent reproductive performance would therefore help determine whether the historical SOI relationship reflects conditions experienced away from the breeding colony.

When the dataset was extended through 2024, support for climatic predictors differed from that observed over the historical period. The nonlinear AGMT relationship received less support (Figure S1), whereas models containing AMO received greater support. This pattern could be consistent with changing climate‐demography relationships under increasingly warm conditions. However, the overlapping temporal windows do not provide a formal test of temporal changes in predictor effects. AMO represents broad, basin‐scale variability in North Atlantic conditions and may integrate oceanographic processes relevant to prey availability and marine productivity. The different support for AGMT, SOI, and AMO across different time periods may therefore reflect shifts in the relationship between broad‐scale climatic conditions and the local foraging environment experienced by Leach's storm‐petrels. Nevertheless, the mechanisms cannot be resolved using broad‐scale climate indices alone. Direct measurements of local oceanographic conditions and prey availability will be necessary to determine whether the temporal patterns identified here correspond to changes in the ecological processes affecting hatching success.

Contrary to our hypothesis, the heatwave index did not improve predictions of hatching success over the period for which this metric was available. Thus, despite the increasing occurrence of extreme marine temperatures, our results provide no evidence that the heatwave metric used here explains interannual variation in hatching success better than broader climatic variability. This does not necessarily indicate that marine heatwaves are not important to Leach's storm‐petrels. Their effects may depend on their timing relative to breeding, their spatial overlap with adult foraging areas, or their influence on prey communities rather than directly on birds. Moreover, relationships with extreme events may become more apparent as marine heatwaves increase in frequency, duration, and intensity (Werner et al. 2026). Future analyses incorporating spatially explicit heatwave exposure within known foraging areas may provide a more direct test of their effects on reproductive performance.

The absence of NAO from the best‐supported models was also unexpected. NAO influences food‐web dynamics in the Gulf of Maine, including the abundance of copepods and their predators (Conversi et al. 2001; Hoogenboom et al. 2015), as well as local wind, precipitation, and temperature conditions that can affect seabird foraging and nest environments (Sandvik et al. 2008; Genovart et al. 2013). We therefore expected NAO to be associated with hatching success of Leach's storm‐petrels breeding in the North Atlantic, yet it was absent from all best‐supported models. This may indicate that NAO is too broad a proxy for the environmental conditions directly affecting reproduction at the colony, or that other environmental and ecological processes are more influential. Factors not included in our models, including precipitation, prey availability, and predation pressure, may also contribute to annual variation in breeding success (Price et al. 2020). Incorporating these more proximal environmental variables should help distinguish the pathways through which broad‐scale climate variability translates into reproductive outcomes.

Climate may also influence reproduction indirectly through demographic processes operating across years. Productivity of seabirds breeding in the North Atlantic is declining (Killeen et al. 2025), and climate effects on survival and breeding behavior could contribute to reproductive responses beyond the immediate effects of environmental conditions during incubation. AMO has been identified as a predictor of adult survival in this population (Pollet et al. 2025), and reduced survival under unfavorable conditions could alter the age and experience structure of the breeding population or disrupt established pair bonds, with subsequent consequences for hatching success. Similarly, increasing AGMT has been associated with higher mate‐switching rates in this species (Pollet et al. 2026), potentially affecting pair coordination and reproductive performance. Climate variability may therefore influence hatching success through multiple pathways, including immediate effects on foraging conditions as well as longer‐term effects on survival, breeding experience, and pair stability. Integrating these demographic processes within a common framework would provide a more complete understanding of how climate affects population dynamics.

More broadly, the temporal sensitivity identified here illustrates an important challenge in using long‐term demographic records to understand climate effects on long‐lived marine vertebrates. Climate–demography relationships need not remain constant as environmental conditions change, because organisms may encounter new climatic regimes, prey communities may reorganize, and the relationships between large‐scale climate indices and local environmental conditions may themselves vary through time. Non‐stationarity in atmospheric circulation and climatic linkages has been documented in the North Atlantic–European sector (Hertig et al. 2015), while changes in climate–biological relationships following oceanographic regime shifts have also been reported in marine ecosystems (Litzow et al. 2018, 2019; Schmidt et al. 2014). Our results cannot establish that such a regime shift occurred in this Leach's storm‐petrel population, but they demonstrate that the climatic relationships inferred from the historical portion of the demographic record are not identical to those supported by the extended time series. Consequently, extrapolating climate–demography relationships estimated from historical periods assumes a degree of stationarity that should be explicitly evaluated when predicting future population responses.

Several limitations should be considered when interpreting these relationships. First, broad‐scale climate indices such as AGMT, AMO, and SOI integrate multiple physical and biological processes and should be regarded as proxies for the environmental mechanisms affecting reproduction rather than direct drivers. In particular, we did not include long‐term information on prey availability, foraging conditions, and adult condition that could identify the pathways linking climate variability to reproductive performance. Second, this study is based on a single breeding colony, and climate–demography relationships may differ among populations experiencing different oceanographic conditions (Hedd et al. 2018). Thirdly, we looked at hatching success, the first part of the reproductive success; we did not evaluate the effects of environmental variables on chick‐rearing and fledgling success. Finally, because candidate environmental models were evaluated separately across overlapping time periods, differences in the best‐supported predictors should not be interpreted as formal evidence that the climatic drivers of hatching success changed through time. Repeated model selection across partially overlapping datasets may increase the opportunity for different predictors to receive support by chance. Thus, the inclusion of SOI in the best‐supported model for 1955–2010 but not 1955–2024 could reflect a temporal change in its association with hatching success, but could also arise from sampling variation and model‐selection uncertainty. The period‐specific analyses were designed to address distinct questions associated with comparison with the historical analysis, extension of the long‐term record, and assessment of the heatwave index over the period for which it was available, rather than to formally identify changes in climate–reproduction relationships through time.

Our study demonstrates that the climatic variables associated with hatching success in Leach's storm‐petrels depend on the temporal extent over which a long‐term demographic record is evaluated. Relationships supported by the historical record were not necessarily maintained when 14 additional years were included, highlighting the potential limitations of assuming stationary climate–demography relationships under ongoing environmental change. For a long‐lived seabird species with conservation concern (BirdLife International 2018), this temporal sensitivity has important implications: projections based solely on historical climate–reproduction relationships may not reliably represent reproductive responses under future conditions. Continued long‐term monitoring, coupled with direct measurements of prey availability, foraging conditions, and adult condition and with comparisons among colonies, will be important for identifying the mechanisms underlying these associations and determining their generality. More broadly, evaluating the temporal stability of climate–demography relationships should improve our ability to anticipate population vulnerability as marine ecosystems continue to warm.

## Author Contributions

**Ingrid L. Pollet:** conceptualization (equal), data curation (lead), formal analysis (lead), investigation (lead), methodology (lead), visualization (lead), writing – original draft (lead), writing – review and editing (lead). **Robert A. Ronconi:** conceptualization (supporting), funding acquisition (lead), investigation (supporting), methodology (supporting), writing – review and editing (supporting). **Robert A. Mauck:** conceptualization (equal), data curation (equal), formal analysis (supporting), investigation (supporting), writing – review and editing (supporting). **Patricia L. Jones:** conceptualization (equal), data curation (equal), investigation (supporting), methodology (supporting), writing – review and editing (supporting).

## Funding

This research was funded by Environment and Climate Change Canada grant GCXE22C311 and GCXE25C037.

## Conflicts of Interest

The authors declare no conflicts of interest.

## Supporting information


**Table S1:** Summary of the all intrinsic models (without climatic variables) of hatching success of Leach's storm‐petrels on Kent Island, for each period tested. AICc, Akaike's information criterion adjusted for sample size; BE, breeding experience; Br, burrow ID; ID, individual ID; K, number of parameters; Pair, Pair ID; PBL, pair bond length; Wt, Akaike weight; Y, year; Δ AICc = difference between model with lowest AICc and other models.
**Figure S1:** Predicted relationship between the Annual Global Mean Temperature (AGMT) and hatching success for Leach's storm‐petrel on Kent Island, New Brunswick based on top performing models for the 1955–2010 dataset (dashed black) and the 1955–2024 dataset (solid blue). Tick marks above the *x*‐axis represent AGMT values during the study period.

## Data Availability

Data and code for this study are available at: https://doi.org/10.6084/m9.figshare.31424723.

## References

[ece374644-bib-0002] Arguez, A. , S. Hurley , A. Inamdar , L. Mahoney , A. Sanchez‐Lugo , and L. Yang . 2020. “Should We Expect Each Year in the Next Decade (2019‐28) to Be Ranked Among the Top 10 Warmest Years Globally?” Bulletin of the American Meteorological Society 101: E655–E663. 10.1175/BAMS-D-19-0215.1.

[ece374644-bib-0003] Bates, D. , M. Maechler , B. Bolker , and S. Walker . 2015. “Fitting Linear Mixed‐Effects Models Using lme4.” Journal of Statistical Software 67: 1–48. 10.18637/jss.v067.i01.

[ece374644-bib-0004] BirdLife International . 2018. “Leach's Storm‐Petrel *Hydrobates leucorhous* Species Factsheet.” BirdLife Data Zone. https://datazone.birdlife.org/species/factsheet/leachs‐storm‐petrel‐hydrobates‐leucorhous.

[ece374644-bib-0005] Burnham, K. P. , D. R. Anderson , and K. P. Burnham . 2002. Model Selection and Multimodel Inference: A Practical Information‐Theoretic Approach. Springer.

[ece374644-bib-0006] Bylhouwer, B. , D. Ianson , and K. Kohfeld . 2013. “Changes in the Onset and Intensity of Wind‐Driven Upwelling and Downwelling Along the North American Pacific Coast.” Journal of Geophysical Research, Oceans 118: 2565–2580. 10.1002/jgrc.20194.

[ece374644-bib-0007] Conversi, A. , S. Piontkovski , and S. Hameed . 2001. “Seasonal and Interannual Dynamics of *Calanus finmarchicus* in the Gulf of Maine (Northeastern US Shelf) With Reference to the North Atlantic Oscillation.” Deep Sea Research Part II 48: 519–530. 10.1016/S0967-0645(00)00088-6.

[ece374644-bib-0008] COSEWIC . 2020. COSEWIC Assessment and Status Report on the Leach's Storm‐Petrel (Atlantic Population) Oceanodroma Leucorhoa in Canada. Committee on the Statius of Endangered Wildlife in Canada Xii + 70 pp. https://www.canada.ca/en/environment‐climate‐change/services/species‐riskpublic‐registry.html.

[ece374644-bib-0063] D'Entremont, K. J. N. , L. M. Zitske , A. J. Gladwell , N. K. Elliott , R. A. Mauck , and R. A. Ronconi . 2020. “Breeding Population Decline and Associations With Nest Site Use of Leach's Storm‐Petrels on Kent Island, New Brunswick From 2001 to 2018.” Avian Conservation and Ecology 15, no. 1: 11. 10.5751/ACE-01526-150111.

[ece374644-bib-0010] Desprez, M. , S. Jenouvrier , C. Barbraud , K. Delord , and H. Weimerskirch . 2018. “Linking Oceanographic Conditions, Migratory Schedules and Foraging Behaviour During the Non‐Breeding Season to Reproductive Performance in a Long‐Lived Seabird.” Functional Ecology 32: 2040–2053. 10.1111/1365-2435.13117.

[ece374644-bib-0011] Dias, M. P. , R. Martin , E. J. Pearmain , et al. 2019. “Threats to Seabirds: A Global Assessment.” Biological Conservation 237: 525–537. 10.1016/j/biocon.2019.06.033.

[ece374644-bib-0012] Fayet, A. L. , G. V. Clucas , T. Anker‐Nilssen , M. Syposz , and E. S. Hansen . 2020. “Local Prey Shortage Drive Foraging Costs and Breeding Success in s Declining Seabird, the Atlantic Puffin.” Journal of Animal Ecology 90: 1152–1164. 10.1111/1365-2656.13442. 33748966

[ece374644-bib-0013] Fennie, H. W. , R. Seary , B. A. Muhling , et al. 2022. “An Anchovy Ecosystem Indicator of Marine Predator Foraging and Reproduction.” Proceedings of the Royal Society B 290: 20222326. 10.1098/rspb.2022.2326. PMC9904941 36750186

[ece374644-bib-0014] Frederiksen, M. , M. P. Harris , F. Daunt , P. Rothery , and S. Wanless . 2004. “Scale‐Dependent Climate Signals Drive Breeding Phenology of Three Seabird Species.” Global Change Biology 10: 1214–1221. 10.1111/j.1529-8817.2003.00794.x.

[ece374644-bib-0015] Genovart, M. , A. Sanz‐Aguillar , A. Fernández‐Chacón , et al. 2013. “Contrasting Effects of Climatic Variability on the Demography of a Trans‐Equatorial Migratory Seabird.” Journal of Animal Ecology 82: 121–130. 10.1111/j.1365-2656.2012.02015.x. 22823099

[ece374644-bib-0016] Gordo, O. , and J. J. Sanz . 2005. “Phenology and Climate Change: A Long‐Term Study in a Mediterranean Locality.” Oecologia 146: 484–495. 10.1007/s00442-005-0240-z. 16170564

[ece374644-bib-0017] Grémillet, D. , and T. Boulinier . 2009. “Spatial Ecology and Conservation of Seabirds Facing Global Climate Change: A Review.” Marine Ecology Progress Series 391: 121–137. 10.3354/meps08212.

[ece374644-bib-0018] Halupka, L. , D. Arlt , J. Tolvanen , et al. 2023. “The Effects of Climate Change on Avian Offspring Production: A Global Meta‐Analysis.” PNAS 120: e2208389120. 10.1073/pnas.2208389120. PMC10175715 37126701

[ece374644-bib-0019] Hansen, J. , M. Sato , R. Ruedy , K. Lo , D. W. Lea , and M. Medina‐Elizade . 2006. “Global Temperature Change.” Proceedings of the National Academy of Sciences of the United States of America 103: 14288–14293. 10.1073/pnas.0606291103. PMC1576294 17001018

[ece374644-bib-0020] Hedd, A. , I. L. Pollet , R. A. Mauck , et al. 2018. “Foraging Areas, Offshore Habitat Use, and Colony Overlap by Incubating Leach's Storm‐Petrels *Oceanodroma leucorhoa* in the Northwest Atlantic.” PLoS One 13, no. 5: e0194389. 10.1371/journal.pone.0194389. PMC5942770 29742124

[ece374644-bib-0064] Hertig, E. , C. Beck , H. Wanner , and J. Jacobeit . 2015. “A Review of Non‐Stationarities in Climate Variability of the Last Century With Focus on the North Atlantic–European Sector.” Earth‐Science Reviews 147: 1–17. 10.1016/j.earscirev.2015.04.009.

[ece374644-bib-0021] Hervé, M. 2017. “RVAideMemoire: Diverse Basic Statistical and Graphical Functions.” https://CRAN.R‐project.org/package=RVAideMemoire.

[ece374644-bib-0022] Hobday, A. J. , L. V. Alexander , S. E. Perkins , et al. 2016. “A Hierarchical Approach to Efining Marine Heatwaves.” Progress in Oceanography 141: 227–238. 10.1016/j.pocean.2015.12.014.

[ece374644-bib-0023] Hoogenboom, J. L. , S. N. Wong , R. A. Ronconi , H. N. Koopman , L. D. Murison , and A. J. Westgate . 2015. “Environmental Predictors and Temporal Patterns of Basking Shark (*Cetorhinus maximus*) Occurrence in the Lower Bay of Fundy, Canada.” Journal of Experimental Marine Biology and Ecology 465: 24–32. 10.1016/j.jembe.2015.01.005.

[ece374644-bib-0024] Infanti, J. M. , and B. P. Kirtman . 2016. “North American Rainfall and Temperature Prediction Response to the Diversity of ENSO.” Climate Dynamics 46: 3007–3023. 10.1007/s00382-015-2749-0.

[ece374644-bib-0025] Jenouvrier, S. 2013. “Impacts of Climate Change on Avian Populations.” Global Change Biology 19: 2036–2057. 10.1111/gcb.12195. 23505016

[ece374644-bib-0026] Jenouvrier, S. , C. Barbraud , and H. Weimerskirch . 2005. “Long‐Term Contrasted Responses to Climate of Two Antarctic Seabird Species.” Ecology 86: 2889–29203. 10.1890/05-0514.

[ece374644-bib-0027] Jenouvrier, S. , J.‐C. Thibault , A. Viallefont , et al. 2009. “Global Climate Patterns Explain Range‐Wide Synchronicity in Survival of a Migratory Bird.” Global Change Biology 15: 268–279. 10.1111/j.1365-2486.2008.01715.x.

[ece374644-bib-0028] Johnson, D. H. 1979. “Estimating Nest Success: The Mayfield Method and an Alternative.” Auk 96: 651–661.

[ece374644-bib-0029] Killeen, H. , W. J. Sydeman , B. Hoover , et al. 2025. “Ecosystems Mediate Climate Impacts on Northern Hemisphere Seabirds.” Communications Earth & Environment 6: 804. 10.1038/s43247-025-02717-z.

[ece374644-bib-0030] Layton‐Matthews, K. , C. E. Regan , M. Ballesteros , et al. 2025. “Demographic Responses of North Atlantic Seabirds to Seasonal Ocean Warming.” Proceedings of the National Academy of Sciences of the United States of America 122: e2507531122. 10.1073/pnas.2507531122. PMC12718362 41359851

[ece374644-bib-0032] Litzow, M. A. , L. Ciannelli , P. Puerta , J. J. Wettstein , R. R. Rykaczewski , and M. Opiekun . 2018. “Non‐Stationary Climate – Salmon Relationships in the Guld of Alaska.” Proceedings of the Royal Society B 285: 20181855. 10.1098/rspb.2018.1855. PMC6235052 30404879

[ece374644-bib-0033] Litzow, M. A. , L. Ciannelli , P. Puerta , J. J. Wettstein , R. R. Rykaczewski , and M. Opiekun . 2019. “Nonstationary Environmental and Community Relationships in the North Pacific Ocean.” Ecology 100: e02760. 10.1002/ecy.2760 31127608

[ece374644-bib-0034] Mauck, R. A. , D. C. Dearborn , and C. E. Huntington . 2018. “Annual Global Mean Temperature Explains Reproductive Success in a Marine Vertebrate From 1955 to 2010.” Global Change Biology 24: 1599–11613. 10.1111/gcb.13982. 29140586

[ece374644-bib-0035] Mauck, R. A. , I. Pratte , A. Hedd , et al. 2022. “Female and Male Leach's Storm Petrels (*Hydrobates leucorhous*) Pursue Different Foraging Strategies During the Incubation Period.” Ibis 165: 161–178. 10.1111/ibi.13112. PMC9798729 36589762

[ece374644-bib-0036] Montevecchi, W. A. , P. M. Regular , J.‐F. Rail , et al. 2020. “Ocean Heat Wave Induces Breeding Failure at the Southern Breeding Limit of the Northern Gannet *Morus bassanus* .” Marine Ornithology 49: 71–78. 10.5038/2074-1235.49.1.1400.

[ece374644-bib-0037] Morley, S. A. , F. Campanella , E. Young , et al. 2025. “Dramatic ENSO Related Southwestern Atlantic Ecosystem Shifts.” Scientific Reports 15: 7917. 10.1038/s41598-025-93080-8. PMC11885653 40050636

[ece374644-bib-0038] Morse, D. H. , and S. W. Kress . 1984. “The Effect of Burrow Loss on Mate Choice in the Leach's Storm‐Petrel.” Auk 101: 158–160.

[ece374644-bib-0039] Oliveira, N. , P. Abreu , H. Alonso , et al. 2023. “The Effect of Environmental Conditions on Captures, Survival and Breeding Success of a Winter‐Breeding Seabirds.” Ardea 111: 487–500. 10.5253/arde.2022.a13.

[ece374644-bib-0041] Pereira, J. M. , V. H. Paiva , F. R. Ceia , and J. A. Ramos . 2020. “Facing Extremes: Cory's Shearwaters Adjust Their Foraging Behaviour Differently in Response to Contrasting Phases of North Atlantic Oscillation.” Regional Environmental Change 20: 77. 10.1007/s10113-020-01662-1.

[ece374644-bib-0042] Pershing, A. J. , M. A. Alexander , C. M. Hernandez , et al. 2015. “Slow Adaptation in the Face of Rapid Warming Leads to Collapse of the Gulf of Maine Cod Fishery.” Science 350: 809–812. 10.1126/science.aac9819. 26516197

[ece374644-bib-0043] Piatt, J. F. , J. K. Parrish , H. M. Renner , et al. 2020. “Extreme Mortality and Reproductive Failure of Common Murres Resulting From the Northeast Pacific Marine Heatwave of 2014–2016.” PLoS One 15: e0226087. 10.1371/journal.pone.0226087. PMC6961838 31940310

[ece374644-bib-0044] Pistorius, P. A. , W. J. Sydeman , Y. Watanuki , S. A. Thompson , and F. Orgeret . 2023. “Climate Change: The Ecological Backdrop of Seabird Conservation.” In Conservation of Marine Birds, edited by L. Young and E. VanderWerf , 245–276. Academic Press.

[ece374644-bib-0045] Pollet, I. L. , A. L. Bond , A. Hedd , C. Huntington , and R. G. Butler . 2019. “Leach's Storm‐Petrel.” In Birds of the World. Cornell Laboratory of Ornithology. 10.2173/bow.lcspet.01.1.

[ece374644-bib-0046] Pollet, I. L. , S. E. Gutowsky , R. A. Ronconi , et al. 2025. “Adult Survival in a Small Seabird, *Hydrobates leucorhous*, Covaries With the Atlantic Multidecadal Oscillation Over the Past Six Decades.” Proceedings of the Royal Society B 292: 20242710. 10.1098/rspb.2024.2710. PMC12187428 40555371

[ece374644-bib-0047] Pollet, I. L. , R. A. Ronconi , M. L. Leonard , and D. Shutler . 2019. “Migration Routes and Stopover Areas of Leach's Storm‐Petrels Oceanodroma Leucorhoa.” Marine Ornithology 47: 55–65. 10.5038/2074-1235.47.1.1293.

[ece374644-bib-0048] Pollet, I. L. , L. U. Taylor , R. A. Mauck , C. E. Huntington , and P. L. Jones . 2026. “Permanent and Temporary Mate‐Switching in a Long‐Lived Seabird: Insights From a 64‐Years Study.” Animal Behaviour 237: 123612. 10.1016/j.anbehav.2026.123612.

[ece374644-bib-0049] Price, C. A. , K. Hartmann , T. J. Emery , E. J. Woehler , C. R. McMahon , and M. A. Hindell . 2020. “Climate Variability and Breeding Parameters of a Transhemispheric Migratory Seabird Over Seven Decades.” Marine Ecology Progress Series 642: 191–205. 10.3354/meps13328.

[ece374644-bib-0050] R Core Team . 2024. R: A Language and Environment for Statistical Computing. R Foundation for Statistical Computing. https://www.R‐project.org.

[ece374644-bib-0051] Raymond, B. , S. A. Shaffer , S. Sokolov , et al. 2010. “Shearwater Foraging in the Southern Ocean: The Roles of Prey Availability and Winds.” PLoS ONE 5: ee10960. 10.1371/journal.pone.0010960. PMC2881033 20532034

[ece374644-bib-0052] Rindorf, A. , S. Wanless , and M. P. Harris . 2000. “Effects of Changes in Sandeel Availability on the Reproductive Output of Seabirds.” Marine Ecology Progress Series 202: 241–252.

[ece374644-bib-0053] Sandvik, H. , T. Coulson , and B.‐E. Sæther . 2008. “A Latitudinal Gradient in Climate Effects on Seabird Demography: Results From Interspecific Analyses.” Global Change Biology 14: 703–713. 10.1111/j.1365-2486.2007.01533.x.

[ece374644-bib-0054] Sandvik, H. , K. E. Erikstad , R. T. Barrett , and N. G. Yoccoz . 2005. “The Effect of Climate on Adult Survival in Five Species of North Atlantic Seabirds.” Journal of Animal Ecology 74: 817–831. 10.1111/j.1365-2656.2005.00981.x.

[ece374644-bib-0055] Schlegel, R. W. , and A. J. Smit . 2018. “heatwaveR: A Central Algorithm for the Detection of Heatwaves and Cold‐Spells.” Journal of Open Source Software 3, no. 27: 821. 10.21105/joss.00821.

[ece374644-bib-0056] Schmidt, A. E. , L. W. Botsford , J. M. Eadie , R. W. Bradley , E. Di Lorenzo , and J. Jahncke . 2014. “Non‐Stationary Seabird Response Reveal Shifting ENSO Dynamics in the Northeast Pacific.” Marine Ecology Progress Series 499: 249–258. 10.3354/meps10629.

[ece374644-bib-0057] Seidov, D. , A. Mishonov , and R. Parsons . 2021. “Recent Warming and Decadal Variability of Gulf of Maine and Slope Water.” Limnology and Oceanography 66: 3472–3488. 10.1002/lno.11892.

[ece374644-bib-0058] Seyer, Y. , G. Gauthier , J. Bêty , J.‐F. Therrien , P. Legagneux , and N. Lecomte . 2023. “Local Food Availability and Nonbreeding Carry‐Over Effects Affect Breeding Propensity and Success of a Tundra‐Nesting Predator, the Long‐Tailed Jaeger.” Ornithology 140: 1–12. 10.1093/ornithology/ukad032.

[ece374644-bib-0059] Sorensen, M. C. , J. M. Hipfner , T. K. Kyser , and D. R. Norris . 2009. “Carry‐Overs Effects in a Pacific Seabird: Stable Isotope Evidence That Pre‐Breeding Diet Quality Influences Reproductive Success.” Journal of Animal Ecology 78: 460–476. 10.1111/j.1365-2656.2008.01492.x. 19021778

[ece374644-bib-0060] Sydeman, W. J. , E. Poloczanska , T. E. Reed , and S. A. Thompson . 2015. “Climate Change and Marine Vertebrates.” Science 350: 772–777. 10.1126/science.aac9874. 26564847

[ece374644-bib-0061] Tavares, D. C. , J. F. Moura , A. Merico , and S. Siciliano . 2020. “Mortality of Seabirds Migrating Across the Tropical Atlantic in Relation to Oceanographic Processes.” Animal Conservation 23: 307–319. 10.1111/acv.12539.

[ece374644-bib-0062] Werner, K. M. , I. Núñez‐Riboni , T. Soltwedel , R. Primicerio , and M. Emblemsvåg . 2026. “Major Heat Wave in the North Atlantic Had Widespread and Lasting Impacts on Marine.” Life. Science Advances 12: eadt125. 10.1126/sciadv.adt7125 PMC12757028 41477824

