# Supplementary material for: Temporal Changes in the Climatic Predictors of Hatching Success in Leach's Storm‐Petrels
Source: Ecol Evol. 2026 Nov 2;16(11):e74644. doi: 10.1002/ece3.74644 (PMC13728331; doi:10.1002/ece3.74644)
Supplement: Supplementary file 1 [file ECE3-16-e74644-s001.docx]

Table S1. Summary of the all intrinsic models (without climatic variables) of hatching success of Leach’s storm-petrels on Kent Island, for each period tested. BE = breeding experience; PBL = pair bond length; Y = year; ID = individual ID; Br = burrow ID; Pair = Pair ID; K = number of parameters; AICc Akaike’s information criterion adjusted for sample size; Δ AICc = difference between model with lowest AICc and other models; Wt = Akaike weight;

| Models for the 1955-2010 period | K | AICc | Δ AICc | Wt |
| --- | --- | --- | --- | --- |
| BE + PBL + (1 \| Year) + (1\|Br) + (1 \| Pair) | 6 | 20584.60 | 0.00 | 0.73 |
| BE + PBL + (1 \| Year) + (1\|Br) + (1\|ID) + (1 \| Pair) | 7 | 20586.62 | 2.01 | 0.27 |
| PBL + (1 \| Year) + (1\|Br) + (1 \| Pair) | 5 | 20594.73 | 10.13 | 0.00 |
| PBL + (1 \| Year) + (1\|Br) + (1\|ID) + (1 \| Pair) | 6 | 20596.75 | 12.14 | 0.00 |
| BE + PBL + (1 \| Year) + (1 \| Pair) | 5 | 20668.94 | 84.34 | 0.00 |
| BE + PBL + (1 \| Year) + (1\|ID) + (1 \| Pair) | 6 | 20670.94 | 86.34 | 0.00 |
| PBL + (1 \| Year) + (1 \| Pair) | 4 | 20681.34 | 96.74 | 0.00 |
| PBL + (1 \| Year) + (1\|ID) + (1 \| Pair) | 5 | 20683.34 | 98.74 | 0.00 |
| BE + PBL + (1\|Br) + (1 \| Pair) | 5 | 20890.10 | 305.50 | 0.00 |
| BE + PBL + (1\|Br) + (1\|ID) + (1 \| Pair) | 6 | 20892.10 | 307.50 | 0.00 |
| PBL + (1\|Br) + (1 \| Pair) | 4 | 20901.17 | 316.57 | 0.00 |
| PBL + (1\|Br) + (1\|ID) + (1 \| Pair) | 5 | 20903.17 | 318.57 | 0.00 |
| BE + PBL + (1 \| Pair) | 4 | 20933.69 | 349.09 | 0.00 |
| BE + PBL + (1\|ID) + (1 \| Pair) | 5 | 20935.69 | 351.09 | 0.00 |
| PBL + (1 \| Pair) | 3 | 20945.43 | 360.83 | 0.00 |
| PBL + (1\|ID) + (1 \| Pair) | 4 | 20947.43 | 362.83 | 0.00 |
| BE + (1 \| Year) + (1\|Br) + (1 \| Pair) | 5 | 22024.84 | 1440.24 | 0.00 |
| BE + (1 \| Year) + (1\|Br) + (1\|ID) + (1 \| Pair) | 6 | 22026.84 | 1442.24 | 0.00 |
| BE + (1 \| Year) + (1 \| Pair) | 4 | 22096.19 | 1511.59 | 0.00 |
| BE + (1 \| Year) + (1\|ID) + (1 \| Pair) | 5 | 22098.19 | 1513.59 | 0.00 |
| BE + (1\|Br) + (1 \| Pair) | 4 | 22370.30 | 1785.69 | 0.00 |
| BE + (1\|Br) + (1\|ID) + (1 \| Pair) | 5 | 22372.30 | 1787.69 | 0.00 |
| BE + (1 \| Pair) | 3 | 22410.77 | 1826.16 | 0.00 |
| BE + (1\|ID) + (1 \| Pair) | 4 | 22412.77 | 1828.16 | 0.00 |
| BE + PBL + (1 \| Year) + (1\|Br) | 5 | 23354.18 | 2769.57 | 0.00 |
| BE + PBL + (1 \| Year) + (1\|Br) + (1\|ID) | 6 | 23356.18 | 2771.57 | 0.00 |
| PBL + (1 \| Year) + (1\|Br) | 4 | 23402.54 | 2817.94 | 0.00 |
| PBL + (1 \| Year) + (1\|Br) + (1\|ID) | 5 | 23404.54 | 2819.94 | 0.00 |
| BE + PBL + (1\|Br) | 4 | 23761.19 | 3176.59 | 0.00 |
| BE + PBL + (1\|Br) + (1\|ID) | 5 | 23763.20 | 3178.59 | 0.00 |
| PBL + (1\|Br) | 3 | 23817.41 | 3232.81 | 0.00 |
| PBL + (1\|Br) + (1\|ID) | 4 | 23819.41 | 3234.81 | 0.00 |
| BE + PBL + (1 \| Year) + (1\|ID) | 5 | 24071.42 | 3486.82 | 0.00 |
| PBL + (1 \| Year) + (1\|ID) | 4 | 24157.30 | 3572.70 | 0.00 |
| BE + PBL + (1 \| Year) | 4 | 24215.15 | 3630.55 | 0.00 |
| PBL + (1 \| Year) | 3 | 24306.45 | 3721.85 | 0.00 |
| BE + PBL + (1\|ID) | 4 | 24477.77 | 3893.17 | 0.00 |
| PBL + (1\|ID) | 3 | 24575.69 | 3991.08 | 0.00 |
| BE + (1 \| Year) + (1\|Br) | 4 | 25065.72 | 4481.11 | 0.00 |
| BE + (1 \| Year) + (1\|Br) + (1\|ID) | 5 | 25067.39 | 4482.78 | 0.00 |
| BE + (1\|Br) | 3 | 25492.44 | 4907.84 | 0.00 |
| BE + (1\|Br) + (1\|ID) | 4 | 25494.03 | 4909.42 | 0.00 |
| BE + (1 \| Year) + (1\|ID) | 4 | 25730.81 | 5146.20 | 0.00 |
| BE + (1 \| Year) | 3 | 25927.97 | 5343.37 | 0.00 |
| BE + (1\|ID) | 3 | 26160.84 | 5576.24 | 0.00 |

| Models for the 1955-2024 period | K | AICc | Δ AICc | Wt |
| --- | --- | --- | --- | --- |
| BE + PBL + (1 \| Year) + (1\|Br) + (1 \| PAIR) | 6 | 22318.68 | 0.00 | 0.73 |
| BE + PBL + (1 \| Year) + (1\|Br) + (1\|ID) + (1 \| PAIR) | 7 | 22320.68 | 2.00 | 0.27 |
| PBL + (1 \| Year) + (1\|Br) + (1 \| PAIR) | 5 | 22328.45 | 9.77 | 0.01 |
| PBL + (1 \| Year) + (1\|Br) + (1\|ID) + (1 \| PAIR) | 6 | 22330.45 | 11.77 | 0.00 |
| BE + PBL + (1 \| Year) + (1 \| PAIR) | 5 | 22404.77 | 86.09 | 0.00 |
| BE + PBL + (1 \| Year) + (1\|ID) + (1 \| PAIR) | 6 | 22406.77 | 88.09 | 0.00 |
| PBL + (1 \| Year) + (1 \| PAIR) | 4 | 22416.07 | 97.38 | 0.00 |
| PBL + (1 \| Year) + (1\|ID) + (1 \| PAIR) | 5 | 22418.07 | 99.38 | 0.00 |
| BE + PBL + (1\|Br) + (1 \| PAIR) | 5 | 22673.51 | 354.83 | 0.00 |
| BE + PBL + (1\|Br) + (1\|ID) + (1 \| PAIR) | 6 | 22675.52 | 356.83 | 0.00 |
| PBL + (1\|Br) + (1 \| PAIR) | 4 | 22680.64 | 361.96 | 0.00 |
| PBL + (1\|Br) + (1\|ID) + (1 \| PAIR) | 5 | 22682.64 | 363.96 | 0.00 |
| BE + PBL + (1 \| PAIR) | 4 | 22713.32 | 394.63 | 0.00 |
| BE + PBL + (1\|ID) + (1 \| PAIR) | 5 | 22715.32 | 396.63 | 0.00 |
| PBL + (1 \| PAIR) | 3 | 22720.41 | 401.72 | 0.00 |
| PBL + (1\|ID) + (1 \| PAIR) | 4 | 22722.41 | 403.72 | 0.00 |
| BE + (1 \| Year) + (1\|Br) + (1 \| PAIR) | 5 | 23757.71 | 1439.03 | 0.00 |
| BE + (1 \| Year) + (1\|Br) + (1\|ID) + (1 \| PAIR) | 6 | 23759.71 | 1441.03 | 0.00 |
| BE + (1 \| Year) + (1 \| PAIR) | 4 | 23830.67 | 1511.99 | 0.00 |
| BE + (1 \| Year) + (1\|ID) + (1 \| PAIR) | 5 | 23832.67 | 1513.99 | 0.00 |
| BE + (1\|Br) + (1 \| PAIR) | 4 | 24162.79 | 1844.11 | 0.00 |
| BE + (1\|Br) + (1\|ID) + (1 \| PAIR) | 5 | 24164.79 | 1846.11 | 0.00 |
| BE + (1 \| PAIR) | 3 | 24201.10 | 1882.41 | 0.00 |
| BE + (1\|ID) + (1 \| PAIR) | 4 | 24203.10 | 1884.41 | 0.00 |
| BE + PBL + (1 \| Year) + (1\|Br) | 5 | 25487.04 | 3168.35 | 0.00 |
| BE + PBL + (1 \| Year) + (1\|Br) + (1\|ID) | 6 | 25489.04 | 3170.36 | 0.00 |
| PBL + (1 \| Year) + (1\|Br) | 4 | 25544.91 | 3226.23 | 0.00 |
| PBL + (1 \| Year) + (1\|Br) + (1\|ID) | 5 | 25546.91 | 3228.23 | 0.00 |
| BE + PBL + (1\|Br) | 4 | 26000.59 | 3681.91 | 0.00 |
| BE + PBL + (1\|Br) + (1\|ID) | 5 | 26002.60 | 3683.91 | 0.00 |
| PBL + (1\|Br) | 3 | 26055.81 | 3737.13 | 0.00 |
| PBL + (1\|Br) + (1\|ID) | 4 | 26057.82 | 3739.13 | 0.00 |
| BE + PBL + (1 \| Year) + (1\|ID) | 5 | 26212.63 | 3893.94 | 0.00 |
| PBL + (1 \| Year) + (1\|ID) | 4 | 26305.68 | 3987.00 | 0.00 |
| BE + PBL + (1 \| Year) | 4 | 26361.27 | 4042.59 | 0.00 |
| PBL + (1 \| Year) | 3 | 26460.49 | 4141.81 | 0.00 |
| BE + PBL + (1\|ID) | 4 | 26723.40 | 4404.72 | 0.00 |
| PBL + (1\|ID) | 3 | 26816.12 | 4497.43 | 0.00 |
| BE + (1 \| Year) + (1\|Br) | 4 | 27219.92 | 4901.24 | 0.00 |
| BE + (1 \| Year) + (1\|Br) + (1\|ID) | 5 | 27220.42 | 4901.74 | 0.00 |
| BE + (1\|Br) + (1\|ID) | 4 | 27775.66 | 5456.98 | 0.00 |
| BE + (1\|Br) | 3 | 27776.16 | 5457.47 | 0.00 |
| BE + (1 \| Year) + (1\|ID) | 4 | 27897.42 | 5578.74 | 0.00 |
| BE + (1 \| Year) | 3 | 28104.87 | 5786.18 | 0.00 |
| BE + (1\|ID) | 3 | 28453.95 | 6135.26 | 0.00 |

| Models for the 1982-2024 period | K | AICc | Δ AICc | Wt |
| --- | --- | --- | --- | --- |
| BE + PBL + (1 \| Year) + (1\|Br) + (1 \| Pair) | 6 | 14525.41 | 0.00 | 0.62 |
| BE + PBL + (1 \| Year) + (1\|Br) + (1\|ID) + (1 \| Pair) | 7 | 14527.41 | 2.00 | 0.23 |
| PBL + (1 \| Year) + (1\|Br) + (1 \| Pair) | 5 | 14528.83 | 3.42 | 0.11 |
| PBL + (1 \| Year) + (1\|Br) + (1\|ID) + (1 \| Pair) | 6 | 14530.83 | 5.42 | 0.04 |
| BE + PBL + (1 \| Year) + (1 \| Pair) | 5 | 14570.52 | 45.11 | 0.00 |
| PBL + (1 \| Year) + (1 \| Pair) | 4 | 14573.31 | 47.90 | 0.00 |
| BE + PBL + (1 \| Year) + (1\|ID) + (1 \| Pair) | 6 | 14574.30 | 48.89 | 0.00 |
| PBL + (1 \| Year) + (1\|ID) + (1 \| Pair) | 5 | 14575.31 | 49.90 | 0.00 |
| PBL + (1\|Br) + (1 \| Pair) | 4 | 14678.69 | 153.28 | 0.00 |
| BE + PBL + (1\|Br) + (1 \| Pair) | 5 | 14680.12 | 154.71 | 0.00 |
| PBL + (1\|Br) + (1\|ID) + (1 \| Pair) | 5 | 14680.69 | 155.28 | 0.00 |
| BE + PBL + (1\|Br) + (1\|ID) + (1 \| Pair) | 6 | 14682.12 | 156.72 | 0.00 |
| PBL + (1 \| Pair) | 3 | 14722.80 | 197.39 | 0.00 |
| BE + PBL + (1 \| Pair) | 4 | 14724.22 | 198.81 | 0.00 |
| PBL + (1\|ID) + (1 \| Pair) | 4 | 14724.80 | 199.39 | 0.00 |
| BE + PBL + (1\|ID) + (1 \| Pair) | 5 | 14726.22 | 200.81 | 0.00 |
| BE + (1 \| Year) + (1\|Br) + (1 \| Pair) | 5 | 15210.16 | 684.76 | 0.00 |
| BE + (1 \| Year) + (1\|Br) + (1\|ID) + (1 \| Pair) | 6 | 15212.17 | 686.76 | 0.00 |
| BE + (1 \| Year) + (1 \| Pair) | 4 | 15257.43 | 732.03 | 0.00 |
| BE + (1 \| Year) + (1\|ID) + (1 \| Pair) | 5 | 15259.43 | 734.03 | 0.00 |
| BE + (1\|Br) + (1 \| Pair) | 4 | 15454.79 | 929.39 | 0.00 |
| BE + (1\|Br) + (1\|ID) + (1 \| Pair) | 5 | 15456.80 | 931.39 | 0.00 |
| BE + (1 \| Pair) | 3 | 15475.41 | 950.00 | 0.00 |
| BE + (1\|ID) + (1 \| Pair) | 4 | 15477.41 | 952.00 | 0.00 |
| BE + PBL + (1 \| Year) + (1\|Br) | 5 | 16479.08 | 1953.67 | 0.00 |
| BE + PBL + (1 \| Year) + (1\|Br) + (1\|ID) | 6 | 16481.08 | 1955.68 | 0.00 |
| PBL + (1 \| Year) + (1\|Br) | 4 | 16507.98 | 1982.58 | 0.00 |
| PBL + (1 \| Year) + (1\|Br) + (1\|ID) | 5 | 16509.99 | 1984.58 | 0.00 |
| BE + PBL + (1\|Br) | 4 | 16831.30 | 2305.89 | 0.00 |
| BE + PBL + (1\|Br) + (1\|ID) | 5 | 16833.28 | 2307.88 | 0.00 |
| PBL + (1\|Br) | 3 | 16856.04 | 2330.63 | 0.00 |
| PBL + (1\|Br) + (1\|ID) | 4 | 16858.04 | 2332.63 | 0.00 |
| BE + PBL + (1 \| Year) + (1\|ID) | 5 | 17145.75 | 2620.34 | 0.00 |
| PBL + (1 \| Year) + (1\|ID) | 4 | 17194.48 | 2669.07 | 0.00 |
| BE + PBL + (1 \| Year) | 4 | 17292.12 | 2766.71 | 0.00 |
| BE + (1 \| Year) + (1\|Br) | 4 | 17305.02 | 2779.61 | 0.00 |
| BE + (1 \| Year) + (1\|Br) + (1\|ID) | 5 | 17307.02 | 2781.61 | 0.00 |
| PBL + (1 \| Year) | 3 | 17347.02 | 2821.61 | 0.00 |
| BE + PBL + (1\|ID) | 4 | 17474.58 | 2949.17 | 0.00 |
| PBL + (1\|ID) | 3 | 17517.18 | 2991.77 | 0.00 |
| BE + (1\|Br) | 3 | 17672.26 | 3146.85 | 0.00 |
| BE + (1\|Br) + (1\|ID) | 4 | 17674.25 | 3148.84 | 0.00 |
| BE + (1 \| Year) + (1\|ID) | 4 | 17965.23 | 3439.82 | 0.00 |
| BE + (1 \| Year) | 3 | 18147.82 | 3622.41 | 0.00 |
| BE + (1\|ID) | 3 | 18314.46 | 3789.05 | 0.00 |

Fig. S1. Predicted relationship between the Annual Global Mean Temperature (AGMT) and hatching success for Leach’s storm-petrel on Kent Island, New Brunswick based on top performing models for the 1955-2010 dataset (dashed black) and the 1955-2024 dataset (solid blue). Tick marks above the x-axis represent AGMT values during the study period.
